# Effects of 15-Days −6° Head-Down Bed Rest on the Attention Bias of Threatening Stimulus

**DOI:** 10.3389/fpsyg.2022.730820

**Published:** 2022-06-27

**Authors:** Shan Jiang, YI-Ming Qian, Yuan Jiang, Zi-Qin Cao, Bing-Mu Xin, Ying-Chun Wang, Bin Wu

**Affiliations:** ^1^Department of Psychology, Beijing Sport University, Beijing, China; ^2^China Astronaut Research and Training Center, Beijing, China; ^3^Engineering Research Center of Human Circadian Rhythm and Sleep, Shenzhen, China; ^4^Space Science and Technology Institute, Shenzhen, China

**Keywords:** head-down bed rest, weightlessness, emotion, attention bias, attention avoidance

## Abstract

Previous researchers have found that head-down bed rest (HDBR) will affect the emotional state of individuals, and negative emotions such as anxiety are closely related to attention bias. The present study adopted the dot-probe task to evaluate the effects of 15-days of −6° HDBR on the attention bias of threatening stimulus in 17 young men, which was completed before (Pre-HDBR), during (HDBR-1, HDBR-8, HDBR-15), after (Post-HDBR) the bed rest. In addition, self-report inventories (State Anxiety Inventory, SAI; Positive Affect and Negative Affect Scale, PANAS) were conducted to record emotional changes. The results showed that the participants’ negative affect scores on HDBR-8 were significantly lower than the HDBR-1 in PANAS while there was no significant difference on positive affect scores and anxiety scores in SAI. And the results showed that at the Pre-HDBR, HDBT-1, HDBR-15, Post-HDBR, the response speed to threatening stimulus was faster than neutral stimulus, but no statistical significance. However, reaction time of threatening stimulus is significantly longer than neutral stimulus in the HDBR-8, indicating that HDBR may have an effect on the participants’ attention bias, and this effect is manifested as attention avoidance.

## Introduction

Attention is an important condition for individual cognitive activities, which acts as a filter in the process of processing external stimuli, and the selective attention distribution is called Attentional bias. When facing neutral stimuli and emotional stimuli at the same time, individuals will assign their attention differently (Mogg et al., 1993), especially showing attention bias to threatening emotions that convey important warning signals (O’Toole and Dennis, 2012; Okon-Singer et al., 2013; Pool et al., 2016). Some researchers have proposed that people have an attention bias toward negative stimuli, especially the attention bias toward threat stimuli, which is considered to be a natural reflex behavior that does not require volitional control, and individuals will detect threat information automatically (Yiend and Mathews, 2001; Constans, 2005). However, there is still ongoing controversy over this point of view. Some researchers hold opposes view on absolute automated processing, and believe that individuals’ handling of threat stimuli is not unconscious, but is affected by a variety of factors (Traczyk et al., 2010). On the other hand, the prevailing consensus is that emotions such as anxiety and fear are closely related to attentional bias (Van Bockstaele et al., 2014). Williams et al. (1997) proposed that highly anxious individuals will allocate more cognitive resources to threatening stimuli in a stressful situation (that is, when state anxiety is high). A large number of studies have found that the susceptibility to negative stimuli is significantly regulated by the individual’s anxiety level, and anxious individuals are more likely to have an attention bias toward threatening stimuli (Muris et al., 2000; Bar-Haim et al., 2010; Armstrong et al., 2012; Van Bockstaele et al., 2014; Shechner and Bar-Haim, 2016).

Conquering the universe is the eternal dream of mankind. With the development of international aerospace engineering entering the space station era, the ability to adapt to a weightless environment is closely related to the successful completion of space mission. The changes in the physiological conditions caused by the head-down bed rest (HDBR) experiment are very similar to the effects of microgravity, and have been used to simulate the physiological and psychological microgravity exposure effects of the human body in the ground flight simulation (Pavy-Le Traon et al., 2007). By restricting physical activity, subjects are fixed at −6° head-down tilt bed to reducing the stimulation caused by gravity. Staying in a head-down bed for a long time will cause body fluids to move up (Thornton et al., 1987), reduce the resistance to gravity, reduce the demand for energy and sensory stimulation, and cause physiological changes such as cardiovascular, muscle, and bone (Scott et al., 2021), leading to cognitive and emotional changes in the autonomic nervous system (Benvenutia et al., 2011).

Similar to other extreme environments, in space, affected by factors such as weightlessness, confined space and circadian rhythm changes, astronauts are prone to negative emotions such as anxiety and depression (Palinkas and Houseal, 2000; Ball et al., 2001). HDBR as a simulation of the space environment, the body is affected by microgravity and environmental constraints, which may induce negative emotions in the subjects and affect their attentional bias toward emotional stimuli. The results of previous studies have found that the subjects’ self-reported negative emotions such as anxiety and depression increase during bed rest (Ishizaki et al., 2002; Chen et al., 2011; Liu et al., 2012), and the subjects’ emotional state may not maintain at the same level for a long time, but fluctuates during the period of bed rest (Qin et al., 2010; Chen et al., 2011). In a 70-day HDBR study, in addition, Basner et al. (2021) found the results of Emotion Recognition Test show subjects favored categories with negative valence over categories with neutral or positive valence in a 60-days HDBR. Therefore, it is reasonable to speculate that the participants’ attention bias to threatening stimuli will change in HDBR.

With the continuous development of manned space technology, the time for astronauts to work in orbit has become longer, the processing of emotional information and emotional regulation are very important to humans (Doré et al., 2016), which affects cognitive abilities such as decision-making, learning and memory. Exploring the changes in the individual’s attentional bias to emotional information in a microgravity environment will help us find suitable countermeasures to improve astronauts’ cognitive and psychological abilities, thereby improving mission performance in space. Surprisingly, a large number of studies have focused on the changes in cognitive function of subjects in HDBR (Rao et al., 2014; Friedl-Werner et al., 2020; Basner et al., 2021), but no research has explored the influence of the weightless environment on the attentional bias of emotional stimuli. We administered the Dot-probe before, during and after the period of HDBR to assess the variation of attentional bias to threatening stimuli. Moreover, participants were given self-report inventories to record their emotional changes at each test point as well.

## Materials and Methods

### Participants

Seventeen healthy men through the social recruitment to participate the experiment, all qualified on basic conditions though the clinical medical examinations and psychological selection, with normal intelligence, and normal vision, no history of mental illness, no psychological disorders, no substance abuse, no organic diseases, genetic diseases or infectious diseases, as well as the underlying physical and psychological abnormality that not suitable for participating this experiment. These participants have a mean age of 27.84 years old (SD *=* 3.03, range: 24–34 years), the mean body mass of the participants was 63.46 kg (SD *=* 5.8, range: 55–77 kg), and their mean height was 168.98 cm (SD = 3.5, range: 163–178 cm).

The participants signed informed consent after a detailed explanation of the study, and obtained a financial award at the end of the study. This study was carried out in accordance with the approval of Space Science and Technology Institute (Shenzhen) Ethics Review Board.

### Procedure

This HDBR was organized by the China Astronaut Research and Training Centre. The current bed rest experiment lasted 27–29 days, which comprised a pre-HDBR period of 7 days, a −6° HDBR period of 15 days to simulate prolonged exposure to weightlessness and a post-HDBR recovery period of 5–7 days. During HDBR, participants were awakened daily at 7:00 AM with lights turned off at 11:00 PM, and must did everything in bed, such as eating, washing, bathing and urinating. During the course of the study, no beverage was provided except pure water, and the type and quantity of food provided to the subjects will be strictly controlled according to the nutritional standards. Participants could watch TV, play games or read, and they could use their mobile phones freely from 8:00 to 10:00 PM every day. There were two people in each room, the temperature of the room was maintained between 22°C and 26°C, and the schedules of daily activities were strictly controlled. Specialized physicians monitored the physiological changes of the subjects, including indicators such as heart rate, respiratory rate, body temperature and weight, and ensured that the physical indicators of the subjects are within the normal range. The whole experiment process was not interrupted, and no subject withdrew from the experiment. The whole experiment was carried out normally according to the plan.

The test time was the fifth day before bed rest (Pre-HDBR), the 1th (HDBR-1), 8th (HDBR-8), 15th (HDBR-15) day during bed rest, and the 5th day after bed rest (Post-HDBR). Each test takes about 20 min. The test time is arranged at 9:30–10:00 in the morning and 14:30–15:00 in the afternoon. Each test of the participants will be conducted in the same time period. Before each test, the Chinese version of State Anxiety Inventory (SAI) and Positive Negative Emotion Scale (PANAS) will be used to assess the emotional state of the subjects during bed rest, and some subjects will be interviewed during bed rest to have a deeper understanding of the subject’s emotional state.

## Attentional Task Stimuli

Emotional facial pictures were used as stimuli to assess the individual’s attention allocation to emotional stimuli. All of the stimulus pictures were monochromatic and had black backgrounds. All of the emotional facial pictures were taken from the Chinese Facial Affective Pictures System (CFAPS, Wang and Luo, 2005). According to the valence and arousal of emotional face pictures, 20 face pictures were selected, including ten angry and ten neutral expressions, half of the male and female faces. In the dimension of valence, mean score of angry face pictures is 2.66 (SD = 0.38) and neutral pictures is 4.52 (SD = 0.50). Mean arousal score is 6.26 (SD = 1.38) for angry face pictures and 3.97 (SD = 0.78) for neutral pictures. There was no difference in brightness and contrast between angry and neutral faces pictures.

In the experiment, angry faces and neutral faces of the same gender appeared in pairs, which were expressed as: anger (left)-neutral (right) and neutral (left)-anger (right), and anger and neutral facial images appear on the left or right side of the gaze cross the same number of times.

### Dot-Probe Task

Dot-probe task is often used to measure attention bias to threatening stimulus (MacLeod et al., 1986; Koster et al., 2005). It is based on the assumption that the response speed to the judgment of the location or nature of the probe stimulus will be faster as the subject pays attention to the area where the subject appears, that is, when the probe stimulus appears in the area that the subject has paid attention to, the response will be faster. Otherwise, it is slow. If the position of the detection point is consistent with the position where the target stimulus appears (valid cues), there is a significant difference between the response time and the response time under inconsistent conditions (invalid cues), indicating that there is an attention bias toward threat stimuli (MacLeod et al., 1986).

**Figure 1:**
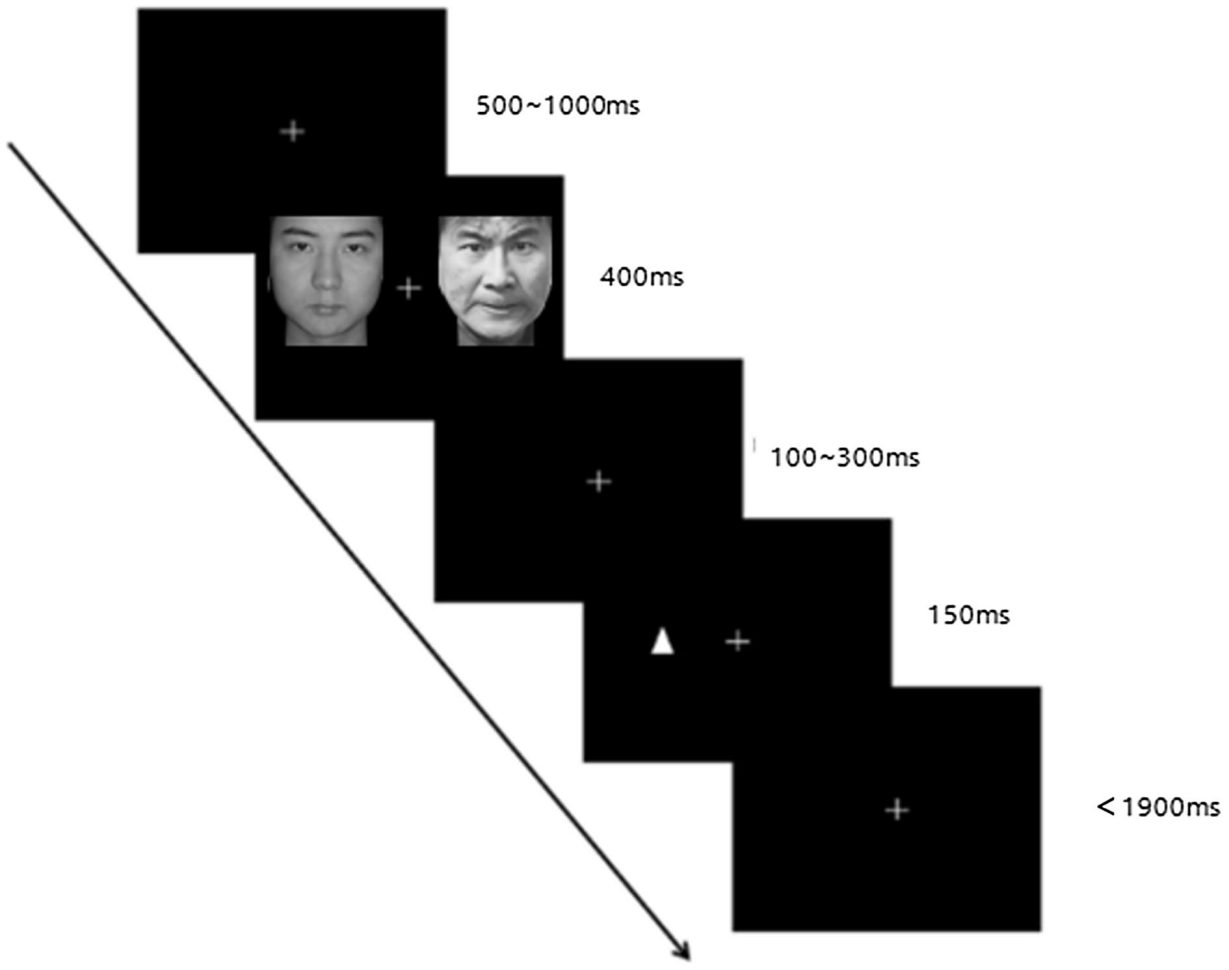
The schematic diagram of the dot-probe task. Reproduced with permission from the Chinese Facial Affective Pictures System (CFAPS).

The E-Prime software program was used to run the dot-probe task, and the process of each trial is shown in Figure 1. First, a white fixation point “+” (1.5 × 1.5 cm) appears in the center of the screen, which randomly appears for 500 ~ 1,000 ms, and then a pair of face pictures appearing on the left and right sides of the screen for 400 ms. After the picture disappears, a random fixation points lasting 100 ~ 300 ms appears “+.” Then a triangular stimulus probe (“Δ” or “∇,” 3 × 3 cm) appears on either side of the picture, and the duration is 150 ms. The interface requires the participants to press the space key as soon as possible and accurately respond to the appearance of the “Δ” symbol (GO trial). After that, the interface automatically skips to the next test; if the “∇” symbol (NO–GO trial time) appears, the subject does not need to make a button response and waits for the next trial. The longest response time is 1900 ms. The proportions of GO and NO–GO trials were 25 and 75%, respectively. There are two blocks in the practice experiment, and each block contains 8 trials. After the end, press any key to automatically enter the formal experiment, including three blocks, each with 64 trials, a total of 192 trials. A 1-min break is arranged in each block. Prior to statistical processing, incorrect responses were deleted and reaction time outliers were filtered using a < 200 and > 1,000 ms cutoff, with subsequent removal of all reaction times (RTs) exceeding 3.0 SD from the a.

### State Anxiety Inventory

The SAI (Spielberger et al., 1983) is comprised of 20 items and usually describes a transient unpleasant emotional experience, such as tension, fear, anxiety, and nervousness, which are generally transient. Participants’ responses on SAI items were provided based on a 4-point scale ranging from 1 representing “nothing at all” to 4 referring to “very obvious,” with a higher score indicating a higher level of anxiety.

### Positive Affect and Negative Affect Scale

The Positive Affect and Negative Affect Scale (PANAS; Huang et al., 2003) is comprised of 20 items and includes two emotional dimensions: positive and negative. Positive emotion factor is composed of 10 adjectives describing positive emotions, and the negative emotion factor is composed of 10 adjectives describing negative emotions. Participants’ responses on PANAS items were provided based on a 5-point scale ranging from 1 representing “almost none” to 5 referring to “extremely much.” High positive emotion scores represent the individual energetic, full attention and in happy emotional state, while low scores indicate indifference. A high negative emotion score indicates confused and painful emotional state, while a low score indicates calmness.

## Statistical Analysis

The Statistical Package for Social Sciences (SPSS 24.0) was used for all analyses. Statistically significant differences were assessed using repeated-measures ANOVAs, the correction was done by Greenhouse–Geisser coefficient. The significance level was set at *p* < 0.05 (two-sided) and partial eta squared $\left(\eta_{p}^{2}\right)$ was presented as the effect size for ANOVA effects. As the results of this experiment all have a correct rate of over 95%, the accuracy rate is not analyzed.

The attention bias was calculated by subtracting the RT of valid cues from the invalid one. Higher scores suggested that there were more biases toward threatening emotional faces. Single-sample *t*-test comparisons were performed to evidence whether bias scores were significantly different from zero.

## Results

### Stimulus Picture Evaluation

In order to verify that the experimental materials also have a good degree of discrimination among the participants in this experiment, after the experiment, the participants were asked to rate the two dimensions of pleasure (1 extremely happy ~ 9 extremely unpleasant) and arousal (1 extremely calm ~ 9 extremely excited) of the material pictures used in the experiment.

The independent-sample T-test was performed to analyze the pleasure and arousal of the threatening face pictures and the neutral face pictures. The results showed that the difference in pleasure was significant, *t*(18) = 10.34, *p* < 0.001; the difference in arousal was significant, *t*(18) = 7.14, *p* < 0.001. The evaluation results are shown in Table 1.

**Table 1:** The Assessment of emotions with 9-point scales (M ± SD, *n* = 17).

	Threatening picture	Neutral picture	*t*	*p*
Valence	3.93 ± 1.29	5.05 ± 0.61	10.34	0.001
Arousal	4.48 ± 2.02	3.53 ± 1.87	7.14	0.001

### Changes in the Emotional Self-Report Inventory Scores of the Participants

**Figure 2:**
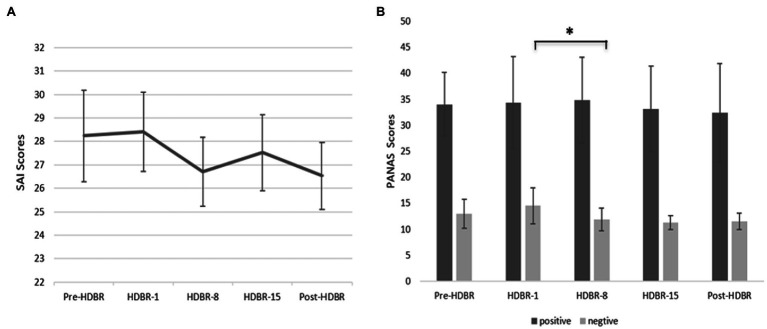
Participants’ State anxiety scores (SAI) and positive affect and negative affect (PANAS) scores at five time points. **(A)** Participants’ performance on SAI at five time points (Pre-HDBR, HDBR-1, HDBR-8, HDBR-15, and Post-HDBR). **(B)** Participants’ performance on PANAS at five time points. Error bars indicate standard deviation of the mean.

**Figure 3:**
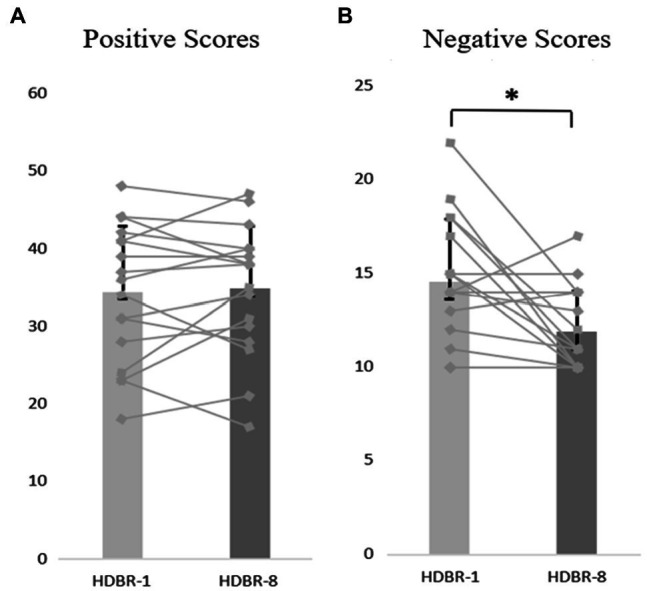
The participants’ individual and mean scores of PANAS at HDBR-1 and HDBR-8, **(A)** Positive Scores. **(B)** Negative Scores. The discount represents the individual’s score, and the column represents the average score. Error bars indicate standard deviation of the mean.

We used a repeated-measure ANOVA to investigate the statistical significance of differences in anxiety, positive and negative affect scores, and the results showed no significant differences in the mean anxiety scores at the different time points (*F*_(4, 64)_ = 0.297, *p* = 0.879, $\eta_{p}^{2}$ = 0.018). These data are depicted in Figure 2. Similarly, there were no significant differences in the positive affect scores at different time points (*F*_(4,64)_ *=* 0.260, *p = 0.831*, $\eta_{p}^{2}$ *=* 0.016). In contrast, results indicated that the main effect of negative affect at different time points was significant (*F*_(4,64)_ = 5.066, *p* < 0.05, $\eta_{p}^{2}$=0.240), but further analysis showed that the negative affect scores on HDBR-8 were significantly lower than the HDBR-1 (*p* < 0.05). These data are depicted in Figures 2, 3.

### Reaction Time

**Figure 4:**
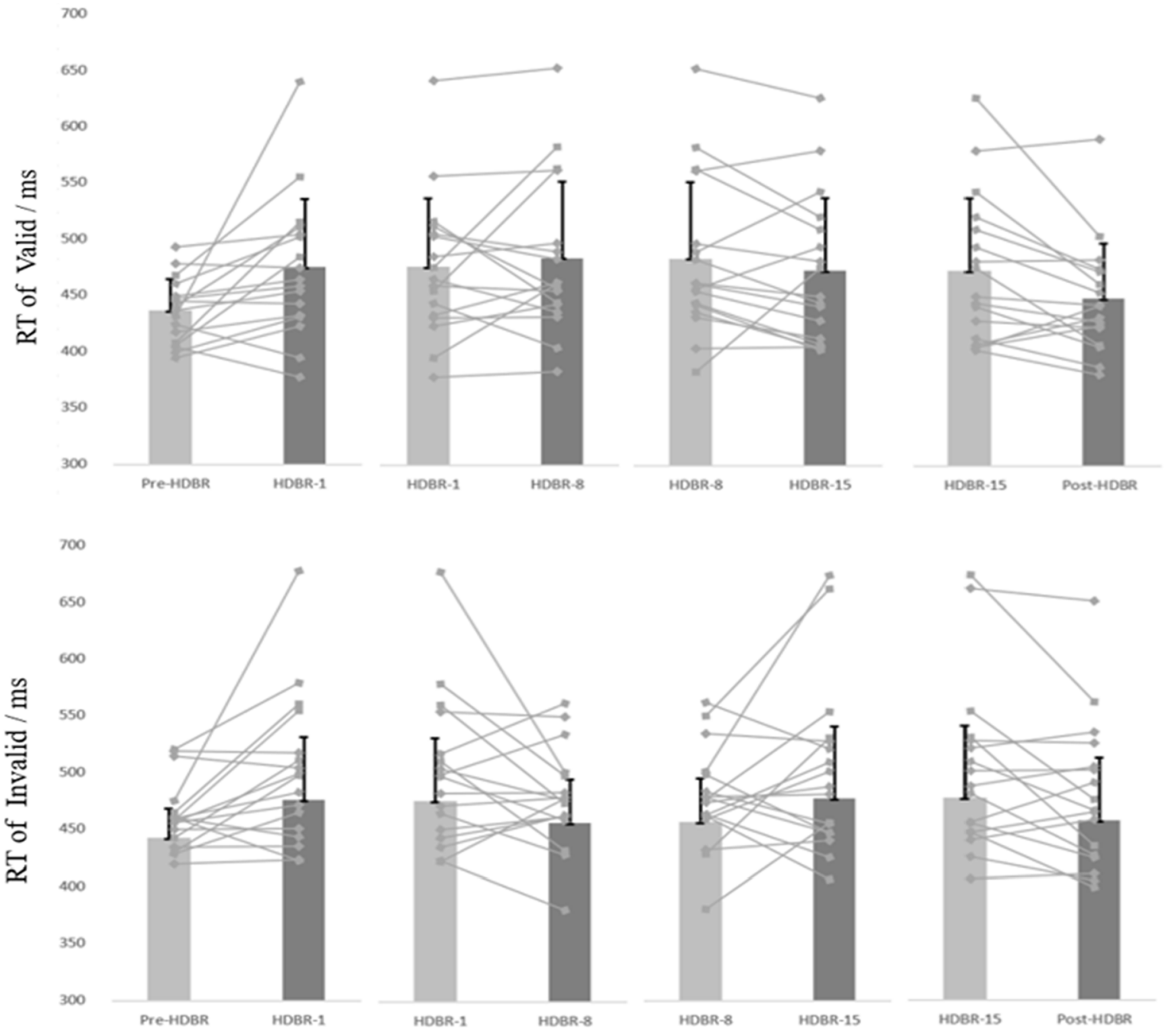
The participants’ individual and mean reaction time of the Dot-probe task at five time points. The discount represents the individual’s score, and the column represents the average score. Error bars indicate standard deviation of the mean.

The participants’ mean reaction time (RT) and attention bias scores in performing the dot-probe task are presented in Table 2. And the participants’ individual RT is presented in Figure 4.

**Table 2:** The Mean reaction time of Dot-probe task (M in milliseconds, *n* = 17).

	Pre-HDBR	HDBR-1	HDBR-8	HDBR-15	Post-HDBR
	M SD	M SD	M SD	M SD	M SD
Valid	435.966	474.730	482.356	471.837	447.379
	28.815	62.534	69.639	66.097	49.627
Invalid	441.950	475.205	455.598	476.798	456.797
	26.712	57.462	38.766	65.483	57.046
Attention bias	5.984	0.475	−26.759	4.960	9.418
	5.105	5.854	11.397	8.120	5.114

For behavioral data, repeated-measures ANOVAs were performed on response time (RT) for the go trials, 2(cue validity: valid, invalid) × 5(time: Pre-HDBR, HDBR-1, HDBR-8, HDBR-15, Post-HDBR). The analysis of RT showed a significant main effect on Time (*F*_(4,64)_ = 4.084, *p <* 0.05, $\eta_{p}^{2}$=0.203), but not Cue validity (*F*_(1,16)_ = 0.186, *p* = 0.672, $\eta_{p}^{2}$=0.011). The response time on the HDBR-1 was significantly longer than the Pre-HDBR (*p* = 0.012), no significant difference on the HDBR-1, HDBR-8 and HDBR-15 (*p* = 0.587; *p* = 0.615), and the reaction time on the Post-HDBR was significantly faster than the HDBR-15 (*p* < 0.05). More importantly, we found a significant Cue validity × Time interaction (*F*_(4,64)_ = 3.51, *p <* 0.05, $\eta_{p}^{2}$ = 0.18). Post-hoc comparisons showed that RT values for the threating stimulus were significantly smaller than those for the neutral (*p* = 0.032) during the HDBR-8, while there was no difference between the two stimuli during other phases.

**Figure 5:**
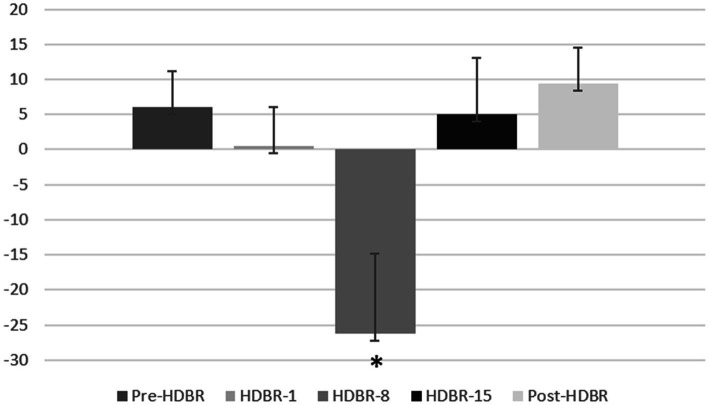
The attention bias scores of the participants at each of five time points. Error bars indicate standard deviation of the mean.

Furthermore, one-sample t-tests on bias scores, in comparison to zero, revealed that HDBR-8 showed attentional avoidance (*t*_(16)_ = −2.397, *p* = 0.029) to threatening stimulus. The mean threatening bias scores at five time point are shown in Figure 5.

## Discussion

Consistent with the expected hypothesis, the results show a significant change in reaction time to valid and invalid cues, indicating that HDBR may affected the male participants’ attention bias. Most importantly in this study, we found that compared with Pre-HDBR, response speed to threat stimuli in the middle of bed rest (HDBR-8) was significantly slower than that of neutral stimuli, attentional avoidance of threatening stimuli appeared.

Cisler et al. (2009) proposed that attention avoidance is to guide attention away from the spatial location of threat cues, which may be an emotion regulation strategy. A large number of studies have found that under extreme stress, attention will divert from threatening stimuli (Bar-Haim et al., 2010; Wald et al., 2011; Shechner et al., 2012). Temporarily turning attention away from threat-related stimuli can reduce the arousal of the sympathetic nervous system, so that individuals can adapt and get used to the environment with painful environment (Gross, 1998). Avoidance of high arousal stimuli may be an adaptive response to overcome the high emotional arousal and excessive aversion caused by strong physical stimuli (Kliemann et al., 2010). When an individual cannot avoid threatening stimulis that require immediate response, attentional avoidance may become an important strategy for regulating emotions such as maintaining current target behaviors or suppressing anxiety (Koster et al., 2005).

But is attention avoidance an appropriate emotion regulation strategy? The vigilance-avoidance hypothesis proposed by Mogg et al. (2004) believes that an anxious individual will initially automatically orient negative information, and then divert attention to avoid the anxious emotional state caused by negative information. This avoidance Strategies may interfere with their objective assessment of these negative information, resulting in individuals still in a state of anxiety. In addition, Shechner and Bar-Haim (2016) proposed a working model of the environment and individual response to threat stimuli. In a dangerous environment, it is vital to improve the vigilance against threat stimuli, which will lead to better adaptive responses and shows the consistency between the situational needs and the level of individual threat monitoring. Wald et al. (2017) conducted attention bias modification (ABM) on soldiers before combat in a study, and the results showed that attention bias training adapted to the environment can alleviate the stress symptoms of soldiers after war. However, if avoidance of threat stimuli occurs in a dangerous environment, it is a bad adaptive response. This kind of attention avoidance does not really divert attention from the high-threat stimulus, but strives to control and no longer pays attention to the threatening stimulus. This kind of effort control is actually an inhibitory mechanism, which can neither promote the correct adaptive response of the individual, but also consume more cognitive resources (Wu et al., 2016). Future research can illustrate the relationship between attention avoidance and cognitive resource consumption by examining the interference of attention avoidance on subsequent cognitive tasks.

Physiological effects may also change attentional bias. The vestibular and emotional brain networks share common subcomponents. (Carmona et al., 2009; Levine et al., 2012). Neuroimaging reveals that vestibular-induced nausea influences the same prefrontal areas of the brain (Miller et al., 1996) that are associated with autonomic regulation of emotions (Demaree and Harrison, 1997). Caloric vestibular stimulation has been shown to influence mood and affective control in healthy participants (Preuss et al., 2014, 2015), and vestibular processing and anxiety networks are functionally intertwined (Besnard et al., 2018). Although gravitational vector input does not change on Earth, the body undergoes axial unloading in the HDBR, simulating the reduced somatosensory inputs in microgravity and this results in sensory reweighting (Moore et al., 2010; Mulavara et al., 2018; Yuan et al., 2018). HDBR does not directly affect vestibular inputs, but sensory reweighting is thought to affect vestibular processing during HDBR. The vestibular nucleus receives the input of sensory information from vestibular organs and limbs (Yates et al., 2000; Jian et al., 2002). During HDBR, the somatosensory receptors distributed throughout the body reduced and the input of foot information is lost, which will affect the vestibular processing (Mulavara et al., 2018). Previous studies have found evidence of sensory reweighting and reduced neural efficiency for vestibular processing in subjects who underwent HDBR intervention (Yuan et al., 2018; Hupfeld et al., 2020). Therefore, we speculate that HDBR will affect the vestibular processing of subjects, thus affecting attentional bias. Thus, as vestibular processing is affected, the ability to feel negative emotions may be disturbed, which affects emotional attentional bias, in addition, because adaptive motor response (fight or flight) and motoric feedback require precise vestibular perception (Preuss et al., 2015), processing vestibular information is closely related to the perception of dangerous and threatening stimuli. As is stated above, individuals will detect threat information automatically (Yiend and Mathews, 2001; Constans, 2005), however may also lag in their perception of threatening stimuli when the functional systems of vestibular is affected. Accordingly, we speculate that HDBR affects the attentional bias of threatening stimuli by affecting vestibular processing. It is worth noting that the above explanation comes from the speculation of previous research theories and it is necessary to prove the relationship between vestibular sensory function and attentional bias through more direct experimental research in future.

Separately examining the participants’ attention bias at other time points, we found that there was no significant difference between valid and invalid cues on RT in the HDBR-15 and Post-HDBR. Similar with other studies, during HDBR, the cognitive function of subjects changed from impairment to recovery (Manzey et al., 1998; Yan et al., 2013). According to the results of this study, we speculate that the change of subjects’ attention bias will not last for a long time, but will change with the change of emotion and environment. In the post HDBR period, through clinical observation and psychological interviews, we learned that subjects have gradually adapted to the head down posture, and vomit, dizziness or other adverse reactions have disappeared. And what is more, the 15th day was the last day of the HDBR, the subjects were in a state of excitement, their attentional bias gradually returned to normal. And from the descriptive statistics, the subjects’ response speed to valid cues is lower than that of invalid cues, and there is a certain degree of attention bias toward threat stimuli. This result may support the predecessor’s view that individual attention is easy to focus on threatening stimuli, and people can quickly detect and respond to threatening information in the environment. This is an innate mechanism of human evolution (Ohman et al., 2001; Putwain et al., 2011).

Inconsistent with some studies (Ishizaki et al., 2002; Chen et al., 2011; Liu et al., 2012), the results of the emotional scale of this study showed that the participant’ negative emotions did not increase significantly during bed rest, even on the eighth day of HDBR, negative scores of PANAS decreased significantly. In a 45 days -6°HDBR study, Xiu et al. (2014) found that the subjects’ anxiety and depression experience during the entire HDBR did not change significantly, but the frontal EEG lateralization index, which represents the ability to regulate emotions, has a significant linear increase, indicating that participants have made a certain effort to regulate their negative emotions to maintain a stable emotional state. In this study, through clinical observations and post-experimental interviews, it was learned that the participants all experienced symptoms of physical anxiety of varying degrees, such as dizziness, waist aches, and insomnia. Therefore, we speculate that the self-rating scale is highly subjective. Although it is convenient, repeated administration of the test may aggravate the subject’s monotonous repetition and present practice effects, increasing the deviation of the measurement results (Wang et al., 2014), and another possibility is that they are very likely to be self-suggested and intentionally conceal their negative emotions. Through interviews, we learned that most of the participants in this study were positive and optimistic, and had the experience of overcoming setbacks, which helped them face negative emotions more actively. However, in view of the lack of support from the results of the emotional scale, whether the subjects’ attentional bias toward threatening stimuli is affected by negative emotional, we still need to be cautious. In future research, it is necessary to adopt more objective method to assess the emotional state of the subjects, and actively explore other factors that affect the individuals’ attention bias. For example, researchers have proposed that general attention ability will affect the attention bias to threat stimuli, and neurocognitive function compromised in a specific domain might affect biased cognition toward an emotional stimulus therein (Hakamata et al., 2016), a few studies have found changes in cognitive performance on neurocognitive function after HDBR (Rao et al., 2014; Yuan et al., 2016), and the functions related to emotions are worthy of our in-depth exploration.

During space missions, humans will endure the complex interplay of psychological and physical stressors. It’s vital to understanding and anticipating that how adaptation to the complex entanglements of physiology, psychology, and behavior could alter an astronaut’s capability to perform an operational task. Excellent and stable attention function is particularly important for astronauts to successfully complete their tasks, the change of attention bias to emotional stimuli may lead individuals to ignore critical information, and the avoidance of threatening stimuli may also consume more cognitive resources. Although the data provided by this study is limited, it has accumulated valuable simulation data for further flight in the orbit, and provided an important reference for selecting and training astronauts.

This study also has certain limitations. One of the primary limitations of this study is the lack of female subjects. This does not provide us with power to evaluate sex differences. In addition, limited by special experimental conditions, eight subjects took traditional Chinese medicine every day during the experiment, which may affect the cognitive function of the subjects. In order to exclude the influence of drug factors on the experimental results, the independent *T*-test was performed on the experimental results (RT) of the two groups of subjects (taking traditional Chinese medicine and no taking), and the result shows that there was no significant difference in response time at the five tests. Although taking Chinese medicine did not affect the results of the experiment, this point should be considered as a limitation. On the other hand, the duration of the experiment may have different effects on individual rhythms and cognitive functions. Short-term space flights that last only one to two weeks will usually not affect the astronauts’ cognitive ability (especially advanced cognitive functions). and mental ability (Shehab et al., 1998). After a longer period of space flight, the individual’s adaptability is exhausted, and new physical and psychological pressures will appear, which will have more complex effects on the astronaut’s psychological and cognitive performance (Liu et al., 2012), attention bias may also change accordingly. A limitation of our study might be that we only performed 15 days of microgravity environment simulation, and it is difficult to predict the possible changes in the long-term microgravity environment. Future studies, should further explore the subjects’ attention bias in the longer-period HDBR.

To our knowledge, this is the first study investigating the effects on attention bias in a 15-day HDBR, and found that in the mid-stage of HDBR, the participants appeared attentional avoidance to threatening stimuli. As a simulation of the space environment, this discovery also provides a certain reference value for the study of the attention bias of the orbiting astronauts. Surviving in space will encounter unforeseen dangers at all times, whether astronauts will show an attention avoidance toward threatening information, and consume more cognitive resources or produce infinite adaptive responses. That is the limitation of this research and it is also the direction of future research.

## Data Availability Statement

The raw data supporting the conclusions of this article will be made available by the authors, without undue reservation.

## Ethics Statement

The studies involving human participants were reviewed and approved by Space Science and Technology Institute (深圳). The patients/participants provided their written informed consent to participate in this study.

## Author Contributions

SJ: study design, data analysis, manuscript draft, and figures. SJ, Y-MQ, B-MX, and Z-QC: data collection. Y-CW, BW, and YJ: manuscript revisions. All authors contributed to the article and approved the submitted version.

## Funding

This research was funded by the grants from the Development and Reform Commission of ShenZhen Municipality (No. XMHT20200104021), the Open Funding Project of National Key Laboratory of Human Factors Engineering (No. SYFD061905K), the Advanced Space Medico-Engineering Research Project of China (No. 2019SY54A0303) and the Comparison of Quick Identification Methods of Negative Emotions in Special Environments (No. 2021SY54B0201).

## Conflict of Interest

The authors declare that the research was conducted in the absence of any commercial or financial relationships that could be construed as a potential conflict of interest.

## Publisher’s Note

All claims expressed in this article are solely those of the authors and do not necessarily represent those of their affiliated organizations, or those of the publisher, the editors and the reviewers. Any product that may be evaluated in this article, or claim that may be made by its manufacturer, is not guaranteed or endorsed by the publisher.
